# A Model Railway System

**Published:** 1909-04-17

**Authors:** 


### The Practitioners' Relaxations.

### A MODEL RAILWAY SYSTEM.

There must be very few boys who do not, at some period or other, take a delight in models, and this delight often remains throughout life. The youngster spends his days on the nursery floor with his toy engines, his boats, and his leaden soldiers. His first engine is doubtless a wooden one, whose motive power is transmitted from its owner through a piece of string. A stage in the advance is shown in the purchase of a small clockwork locomotive, with perhaps a circle of tin rails for it to pursue its brief career on. Then, if the interest in things mechanical is still maintained as the youngster's age gets near double figures, and he is importunate on the subject, the parents may succumb to the "summation of stimuli" and buy a "real steam engine." This at first is carefully kept in its box all day till father comes home. Father is not allowed to sit long after his dinner, the cloth is hurriedly cleared away, and "poor father" is soon hard at work, with one eye on the pressure gauge and the other on the boy.

The next stage is marked by the boy being allowed to raise steam for himself without parental supervision, and from that time his progress in the model line is rapid and is only controlled by the depth of the parental purse and to some extent by the amount of interest "father" himself exhibits in models. A delight in models is often seen in adults whose calling is far removed from anything of a mechanical nature. I have often noticed this in the pages of "The Model Engineer," where I have seen descriptions of scale model locomotives made by a hotel servant, a plumber, an architect's draughtsman, and a sergeant in the Marines; a fine model engine and boiler made out of scrap metal by a mason's labourer; a splendid model traction engine constructed by a bricklayer; and a model sailing yacht made by a blacksmith.

Our own profession is not such an unmechanical one as some I have mentioned. A surgeon is trained to use many and various instruments with accuracy of eye and delicacy of touch, so that, given the interest, a surgeon ought to be a good model-maker.

One of the finest model locomotives ever made is the scale model of the L. B. and S. C. R. locomotive "Como," constructed by a member of the medical profession, while two brothers, also medical men, made, some ten years ago, a most interesting miniature representation of the L. B. and S. C. R. system from London Bridge and Victoria down to Croydon. Their locomotives were of clockwork.

My own particular hobby lies in the same direction. For the last two years I have occupied my spare time in constructing a model railway in a large spare room upstairs. The line is supported on trestles round the room, and at first the rails were of tin and the locomotives were steam ones. These necessitated a considerable amount of running after to stop when required, to say nothing of burnt fingers or the danger of a flare-up when an accident happened and the loco upset. So the steam locomotives and tin rail were done away with. Now the track is equipped with proper scale model brass rail with wooden sleepers, chairs, and keys, while the locomotives, which look exactly like steam ones, are really electric. They can be controlled entirely from the signal-box, and all starting, stopping, reversing, and shunting can now be done without leaving the control levers.

Certainly from a model-making point of view the construction of a model railway offers the greatest diversity of work to the model-maker. First, he is a surveyor, marking out the garden or the room for the future line, and planning the railway to suit the available space. Then he is a navvy, making the foundations for the railway. Then he joins the Permanent Way Department, and lays the sleepers and rails, fastens down chairs, hammers in keys, constructs points and crossings. The signal-box next claims his attention with its levers for points and signals, its interlocking frame, and its connections to be made to signal-posts and points. Then if he is going in for electric locomotives, he must assume the duties of electrician and work out the electric connections used in conjunction with the signalling apparatus. Then he blossoms out as an architect and designs stations, engine sheds, warehouses, and tunnels. By the time he has built these he has become a more or less successful woodworker, and to finish them off he must assume the rôle of a glazier, painter, and house decorator. Then if he makes his own locomotives and rolling stock the list is still further increased. No other model makes its constructor follow such a variety of trades as does a model railway. As time goes on, and the railway system develops, it is possible to add many accessory buildings, such as a colliery with its pit-head gear and winding-house, a dock with its cranes, and a miniature power-station in which the model railway company can make its own electric current for traction and for lighting its stations and goods yards. There is enough in a complete model railway system to keep its constructor busy for several years; in fact, until "the boys" are old enough to manage and look after it for themselves.

